# The relationship between measures of foot mobility and subtalar joint stiffness using vibration energy with color Doppler imaging-A clinical proof-of-concept validation study

**DOI:** 10.1371/journal.pone.0237634

**Published:** 2020-08-19

**Authors:** Mark P. Wilhelm, Troy L. Hooper, Gesine H. Seeber, Kevin L. Browne, Elizabeth Sargent, Kerry K. Gilbert, C. Roger James, Jean-Michel Brismée, Omer C. Matthijs, Anja Matthijs, Phillip S. Sizer

**Affiliations:** 1 Tufts University School of Medicine, Medford, Massachusetts, United States of America; 2 Center for Rehabilitation Research, Texas Tech University Health Sciences Center, Lubbock, Texas, United States of America; 3 University Hospital for Orthopaedics and Trauma Surgery Pius-Hospital, Medical Campus University of Oldenburg, Oldenburg, Germany; 4 College of Health Sciences, University of Texas at El Paso, El Paso, Texas, United States of America; 5 Department of Rehabilitation and Movement Science, University of Vermont, Burlington, Vermont, United States of America; 6 Texas Tech University Health Sciences Center, Lubbock, Texas, United States of America; 7 Boma, Physical Therapy Outpatient Clinic, Kapfenberg, Styria, Austria; Toronto Rehabilitation Institute - UHN, CANADA

## Abstract

**Introduction:**

Subtalar joint (STJ) dysfunction can contribute to movement disturbances. Vibration energy with color Doppler imaging (VECDI) may be useful for detecting STJ stiffness changes.

**Objectives:**

(1) Support proof-of-concept that VECDI could detect STJ stiffness differences; (2) Establish STJ stiffness range in asymptomatic volunteers; (3) Examine relationships between STJ stiffness and foot mobility; and (4) Assess VECDI precision and reliability for examining STJ stiffness.

**Methods:**

After establishing cadaveric testing model proof-of-concept, STJ stiffness (threshold units, ΔTU), ankle complex passive range-of-motion (PROM) and midfoot-width-difference (MFWDiff) data were collected in 28 asymptomatic subjects *in vivo*. Three reliability measurements were collected per variable; Rater-1 collected on all subjects and rater-2 on the first ten subjects. Subjects were classified into three STJ stiffness groups.

**Results:**

Cadaveric VECDI measurement intra-rater reliability was 0.80. A significantly lower STJ ΔTU (p = .002) and ankle complex PROM (p < .001) was observed during the screw fixation versus normal condition. A fair correlation (r = 0.660) was observed between cadaveric ΔTU and ankle complex PROM. *In vivo* VECDI measurements demonstrated good intra-rater (0.76–0.84) versus poor inter-rater (-3.11) reliability. Significant positive correlations were found between STJ stiffness and both dorsum (*r* = .440) and posterior (*r* = .390) PROM. MFWDiff exhibited poor relationships with stiffness (*r* = .103) and either dorsum (r = .256) or posterior (r = .301) PROM. STJ stiffness ranged from 2.33 to 7.50 ΔTUs, categorizing subjects’ STJ stiffness as increased (n = 6), normal (n = 15), or decreased (n = 7). Significant ANOVA main effects for classification were found based on ΔTU (p< .001), dorsum PROM (p = .017), and posterior PROM (p = .036). Post-hoc tests revealed significant: (1) ΔTU differences between all stiffness groups (p < .001); (2) dorsum PROM differences between the increased versus normal (p = .044) and decreased (p = .017) stiffness groups; and (3) posterior PROM differences between the increased versus decreased stiffness groups (p = .044). A good relationship was found between STJ stiffness and dorsum PROM in the increased stiffness group (*r* = .853) versus poor, nonsignificant relationships in the normal (r = -.042) or decreased stiffness (r = -.014) groups.

**Conclusion:**

PROM may not clinically explain all aspects of joint mobility. Joint VECDI stiffness assessment should be considered as a complimentary measurement technique.

## Introduction

Throughout the gait cycle, the subtalar joint (STJ) acts as a shock absorber and assists in propulsion [1]. This joint develops movement dysfunction, exhibiting increased joint stiffness and or instability [2–5]. Other joints in the kinematic chain react with aberrant biomechanical responses, pain, further dysfunction, and disability [4–7].

Studies assessing the relationship between STJ dysfunction and foot mobility are limited in number and scope. Calcaneal fractures have been found to display altered joint kinematics [8,9] where resulting STJ dysfunction is related to both post-fracture immobilization and internal fixation devices [5,10]. This leads to increased lateral foot loading and altered plantar pressure patterns [5]. Additionally, STJ dysfunction can result from primary and or secondary arthritis [10] and diabetes mellitus [2,3,11,12].

Few approaches to STJ mobility assessment have been reported. Loaded and unloaded plain-film stress radiographic imaging can speculate STJ dysfunction, where larger foot posture differences correspond with clinical instability [13]. Diagnostic ultrasound, computerized tomography, and magnetic resonance imaging can provide important information concerning anatomical tissue integrity [13–15]. However, these provide limited objective STJ mobility data, as well as mechanical tissue properties (elasticity, stiffness or strain response).

Dorsal arch height difference, midfoot-width-difference (MFWDiff) and foot mobility magnitude represent recently reported foot mobility measures [16]. The MFWDiff demonstrates a good relationship between static posture and midfoot mobility [16]. However, it does not specifically examine STJ mobility, nor assess its relationship with midfoot mobility. Additionally, static foot posture measures may neither represent dynamic mobility changes [17,13] nor foot function during dynamic conditions [17,18]. Three-dimensional, laboratory kinematic assessment models have been used for STJ mobility testing through: (1) cadaveric STJ movement simulators; and (2) *in vivo* skin-mounted infra-red markers overlying bony structures [2,19]. However, such methods may be cost- and time-prohibitive for clinical mobility testing.

Clinical STJ mobility examination typically consists of clinicians’ qualitative, hands-on articular mobility assessments [20], painful ligament palpations and integrity testing [13], and goniometric joint angle measurements [21] that only grossly assess STJ motion and exhibit appreciable inherent error. The medial subtalar glide test and the medial talar tilt test demonstrate moderate diagnostic accuracy when grossly assessing STJ mobility [20]. However, no clinical examination tests exist to objectively assess for decreased STJ mobility or stiffness changes [22]. In response, an objective and quantitative STJ mobility assessment method is needed.

Joint movement (mobility) occurs in a rotatory fashion around an axis and translatory fashion along a plane. Because direct STJ mobility measurement is prohibited by structural complexity and challenged by clinical technique differences, joint stiffness measurements may serve as a quantifiable representation of joint mobility and dysfunction. Joint stiffness, when mechanically defined, represents the joint’s resistance to movement in the absence of overall range-of-motion measures [23,24]. Joint stiffness, is defined in the current investigation as the amount of vibration energy transferred from one bone (e.g. talus) across a joint (e.g. STJ) to a second bone (e.g. calcaneus), serving to represent clinical STJ mobility status. A joint with greater vibration energy transfer from one bone to another can be said to exhibit greater stiffness due to energy conduction across inflexible tissues that are prone to vibrate. Thus, it is expected that this increased stiffness would be accompanied by reduced joint mobility.

Vibration energy with color Doppler imaging (VECDI) appears in different studies as a responsive joint stiffness measurement when range-of-motion and joint mobility are difficult to assess, such as in the sacroiliac joint [24–28] and first tarsometatarsal joint [23,29]. The VECDI measures joint stiffness by applying high frequency and low amplitude vibration to one joint’s bone (here, calcaneus). The vibration transfers through the joint soft tissue, causing the joint’s other bone (here, talus) to simultaneously vibrate. The amount of vibration energy that is attenuated as it traverses the joint is inversely proportional to the joint’s stiffness [28]. As color Doppler imaging measures the attenuation, the Doppler instrument sensitivity is changed via a threshold unit (TU) setting. The setting determines the sensitivity threshold at which vibration in each bone across the joint is no longer detectible. The difference in the sensitivity threshold represents joint stiffness (expressed as ΔTU), where a small difference (i.e. small ΔTU) between the two bones represents a stiff joint, while a larger difference (i.e. larger ΔTU) represents a less stiff joint. Because the vibration amplitudes are small (<0.05mm) and occur with the joint in resting position, this measurement cannot be said to measure joint mobility but rather measures a joint stiffness construct. Using VECDI to measure STJ stiffness could serve as a reference standard for validating clinical qualitative STJ mobility measurement. Moreover, it could be used to quantify STJ stiffness and examine relationships between stiffness and patient complaints. Finally, it could be used to measure STJ stiffness changes in response to therapeutic manipulative intervention.

Before VECDI can be applied to individuals with pathology, it is important to describe the range of values that occur in asymptomatic people and assess the relationship between STJ stiffness and foot mobility. Such results could suggest the role of STJ stiffness measurement for understanding STJ mobility. However, no studies to date have used VECDI to measure STJ stiffness in an asymptomatic population, nor have any investigators reported VECDI intra- and inter-rater reliability. Our study’s purpose was to establish STJ stiffness ranges in asymptomatic volunteers, support proof-of-concept that VECDI could detect STJ stiffness differences and examine relationships between STJ stiffness and foot mobility. Additionally, we aimed to assess precision and both intra-rater and inter-rater reliability of VECDI for examining STJ stiffness *in vivo*. This study’s results could justify future investigations for using VECDI to further understand ankle-foot mechanical properties, as well as to examine diagnostic and clinical accuracy of STJ testing and treatment approaches. Moreover, by establishing the relationship between STJ laxity and MFWDiff, we intended to understand connections between STJ stiffness and foot function.

In order to address the study purposes, we first aimed to establish VECDI intra- and inter-rater reliability when assessing STJ stiffness *in vivo*. We hypothesized that testers would demonstrate good VECDI intra- and inter-rater reliability (Hypothesis 1 / H1). Next, we aimed to examine relationships between STJ VECDI-derived values and functional STJ inversion and eversion mobility measures (passive range-of-motion [PROM]) using an electrogoniometer (ELGON); as well as MFWDiff. We hypothesized that a good to excellent correlation would be observed between STJ stiffness (i.e. ΔTU) and both PROM and MFWDiff (Hypothesis 2 / H2). Finally, we asked how VECDI-derived values could be used to classify an individual as having decreased, normal or increased STJ stiffness and how that classification could influence the previously described relationships. We hypothesized that different relationships would be observed within the different classification groups, based on stiffness differences across the groups (Hypothesis 3 / H3).

## Materials and methods

### Ethical considerations

All cadavers used in this study were obtained from the Willed Body Program at the Institute of Anatomical Sciences of the Texas Tech University Health Sciences Center (TTUHSC). None of the cadavers were from a vulnerable population and all donors or next kin provided written informed consent that was freely given. All cadaveric specimens were handled in accordance with TTUHSC policies and regulations as determined by the Texas State Anatomical Board. All human subjects’ *in vivo* data collection was approved prior to initiation by the TTUHSC Lubbock/Odessa Institutional Review Board for Protection of Human Subjects (#-L14-036). Informed consent was obtained from each participant in accordance with TTUHSC Lubbock/Odessa Institutional Review Board for Protection of Human Subjects’ policies and procedures. In addition, all procedures followed the Helsinki Declaration’s ethical principles.

**Fig 1:**
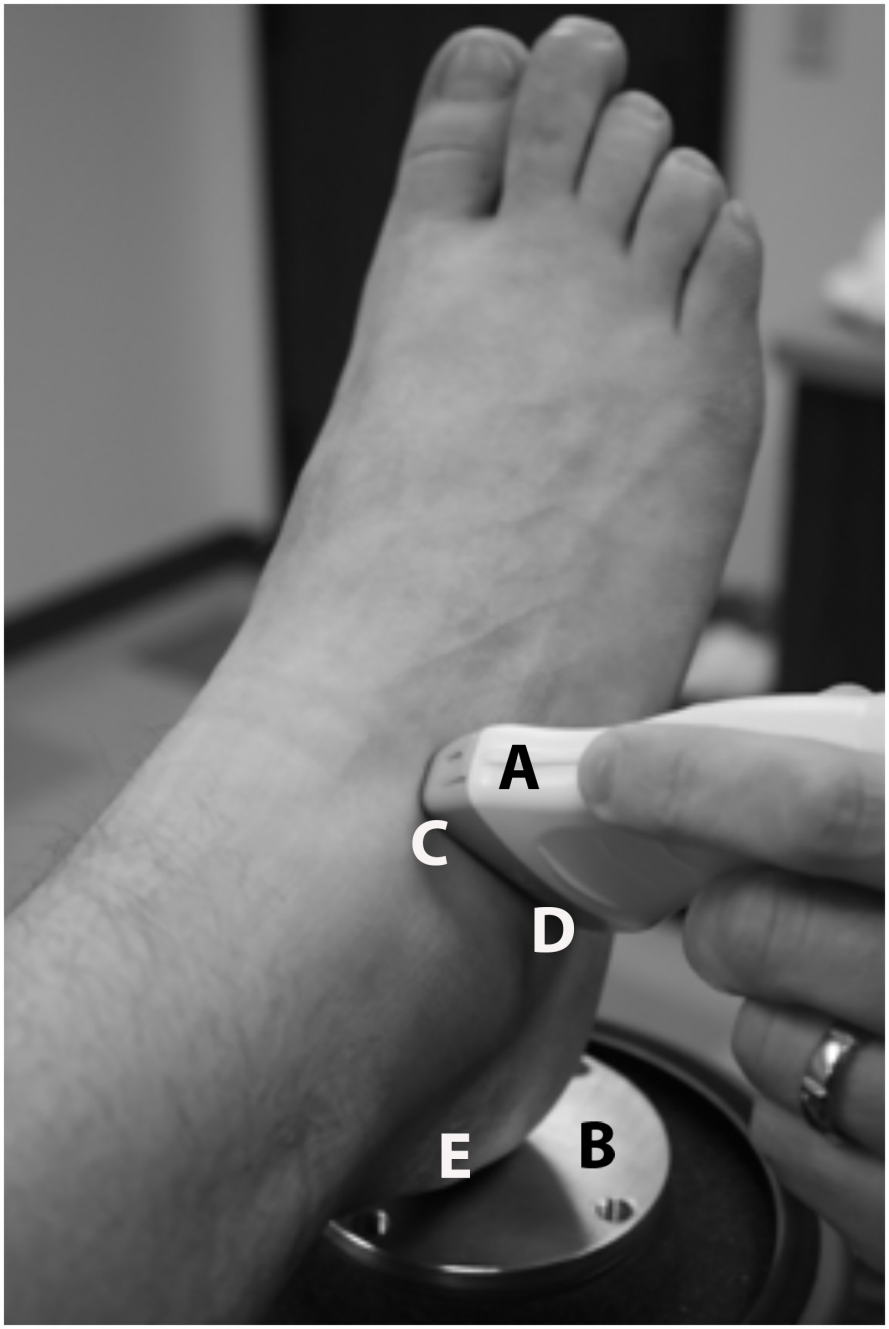
Sound head placement on the lateral STJ. “A = Ultrasound probe; B = Vibration generator platform; C = End of ultrasound probe positioned over the lateral talar neck; D = End of ultrasound probe positioned over the distal lateral calcaneus; E = Calcaneal point of contact over the vibration generator”.

Moreover, the individuals in this manuscript (Fig 1) have given written informed consent to publish their case details.

### Preliminary cadaveric testing

Prior to VECDI testing *in vivo*, eight un-embalmed cadaveric STJ’s were used for preliminary testing. Such testing was meant to test if the planned study procedures could be transferred to an *in vivo* investigation described below. To determine if VECDI could detect STJ stiffness changes, measurements were completed before and after STJ fixation using two 7mm cannulated surgical screws (CSS-900 MaxTorque System, Orthohelix Surgical Design, Inc.; Medina, OH, USA) in a delta configuration. Such surgical screw delta configuration produces the greatest STJ stability [30]. Prior to VECDI testing, ankle-foot complex PROM was measured during both cadaveric conditions (before versus after STJ screw fixation) using the following procedure, in order to confirm joint mobility differences between a stabilized versus un-stabilized cadaveric condition.

Prior to VECDI testing, two ELGONs were used to collect cadaveric ankle-foot complex PROM. One ELGON was applied to the ankle-foot’s dorsum spanning from the distal tibia to the second metatarsal. A second ELGON was adhered on the ankle complex’s posterior aspect from a level approximately 5cm proximal to the lateral malleolus to the posterior distal calcaneus. Ankle-foot inversion / eversion PROM values were collected by passively moving the calcaneus to joint end range three times, where each of the ELGONs measured a different joint system–STJ and midfoot inversion / eversion, respectively.

Investigators then removed the ELGONs and the table was lowered in order to contact the posterior calcaneus on the top plate of the vibration energy shaker apparatus (Labworks Inc. ET-139 75lb shaker. Costa Mesa, California) while sustaining the 10 degrees of ankle plantar flexion (Fig 1). Vibration energy from the shaker device was introduced to the calcaneus at a high frequency (200 Hz) and low amplitude (<0.05mm). A two-pound cuff weight was then placed on the distal anterior tibia to further standardize calcaneal contact.

**Fig 2:**
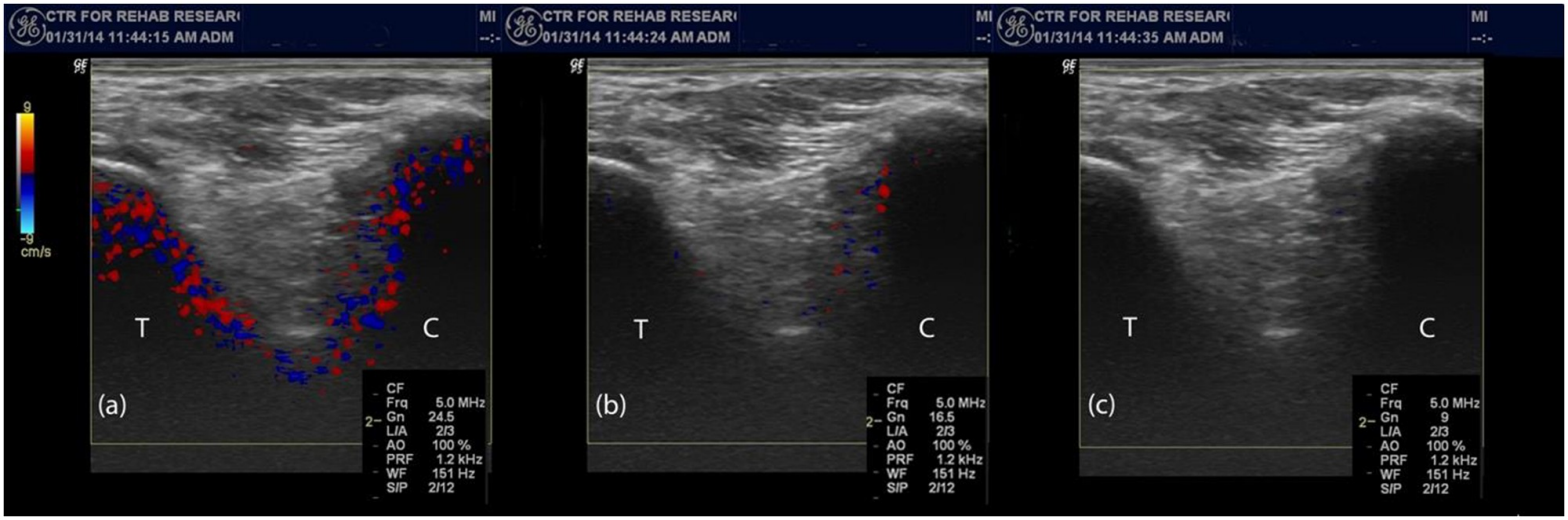
Color Doppler images of the STJ from the lateral side in three different levels of Threshold Unit (TU) settings. (a) with TU setting high, vibration detected on both talus and calcaneus as recognizable by color pixels on both talus and calcaneus; (b) vibration no longer detected in talus = TU_talus_; as recognizable by missing color pixels on talus; (c) vibration no longer detected in calcaneus = TU_calcaneus_: as recognizable by missing color pixels on calcaneus: T = talus and C = calcaneus.

Vibration energy was detected by an ultrasound transducer (General Electric LOGIQ P5. GE Healthcare. Milwaukee, Wisconsin) spanning from the talar neck to the distal lateral calcaneus (Fig 1). Vibration energy was presented on the color Doppler monitor as pixels (red and blue), where the intensity of vibration pixels from the calcaneus and talus appeared simultaneously on the monitor at sufficiently high threshold values (Fig 2). The minimum threshold unit (TU; dimension dB) levels were then determined for both the calcaneus and talus by lowering the color Doppler image registry to the level that vibration from each structure was minimally detectable. At a certain TU value, both the calcaneus and talus vibrated with energy sufficient to display color pixels on the monitor. The TU level was then decreased until the sensitivity of the Doppler fell below the color threshold on the screen for each structure (TU_talus_ vs. TU_calcaneus_, respectively). The calcaneal threshold units (TU_calcaneus_) were subtracted from the talar threshold units (TU_talus_), resulting in the difference in vibration threshold at the two boney structures (ΔTU). Such ΔTU values were used as a measure of STJ stiffness.

This procedure for measuring ΔTU was repeated two additional times and a mean value was calculated for analysis. In order to begin addressing H1 using cadaveric specimens, one rater performed all TU measures three times each. Once a measurement was taken, the US probe was removed for 10 seconds before it was again placed in the same position on the cadaver’s foot and the next measurement was taken. No other factors were adjusted between the measurements. The rater was a physical therapist with 4 years of musculoskeletal ultrasound experience.

### *In vivo* testing

*A priori* defined criteria of success and, thus, justification for conducting an *in vivo* follow-up study were (1) VECDI intra-rater reliability of at least 0.75 and (2) statistically significant lower ΔTU and ankle-foot PROM values, respectively, following cadaveric STJ screw fixation compared to the unscrewed condition. Because these criteria were met (see Results) in the cadaveric study of this investigation, the *in vivo* follow-up study was justified.

Based on a moderate effect size (d = 0.5), an alpha level of .05 and 80% power, a sample size of 28 was required for final *in vivo* data analysis [31]. Thus, for the *in vivo* investigation, a convenience sample of 28 asymptomatic subjects (18–50 years old) was used. Exclusion criteria were: 1) known, unhealed calcaneus or talus fractures; 2) known tumor in the ankle-foot bony structures; 3) current formal healthcare for a lower extremity injury; 4) open wounds in the ankle-foot region; and 5) inability of the investigators to discriminate VECDI readings between the subject’s talus and calcaneus. A history of lower extremity injuries did not exclude subjects from participation if the subject did not present with current pain at the time of data collection.

Prior to *in vivo* data collection, all subjects signed an approved informed consent form. Next, the subject’s dominant weight-bearing leg, defined as the leg that the subject would stand on while kicking a ball, was identified. The subject was asked to lie supine on a high/low table. The dominant leg was rested in a custom-made jig designed to position the test lower extremity in approximately 70 degrees of hip and knee flexion. The ankle was positioned at approximately 10 degrees of plantar flexion for all testing procedures. The subject's non-dominant limb was placed in a hook-lying position and the hands were placed in a resting position at the subject's side. In order to address H1 for the *in vivo* subjects, two separate raters performed TU measures three times each using the VECDI procedure as previously described. No other factors were adjusted between the measurements. Each of the two raters was blinded to the other rater’s TU values, their own TU values, and the PROM values. Both raters performed all TU measurements on the first 10 subjects while the second rater exclusively performed all TU measurements for the remaining 18 subjects. Both raters were physical therapists with musculoskeletal ultrasound experience (one was the same as in the cadaveric study and the other with 2 years of experience).

In order to address H2, subjects were instructed to stand on the ground with both shoes and socks removed and feet positioned approximately shoulder-width apart with an estimated even weight distribution between the lower extremities. A digital caliper (Fowler Xtra-Range 0-600mm Digital Caliper) was used to measure foot width three times at the mid-point (50% of foot length) in the standing position. The subject was then asked to sit on the high/low table with both feet suspended off the ground. A lightweight rigid platform was raised to the plantar surface of the subject’s foot with care to not deform the foot’s resting position. Three additional foot width measurements were performed in this position. For all midfoot width measurements, the measuring rater was blinded to the measurement values. Midfoot-width-difference was calculated by subtracting the non-weight bearing width from the weight bearing width for each subject and the resulting values were used as a measure of foot mobility.

### Data analyses

Data analysis was completed using IBM SPSS version 21.0 for Windows (IBM Corp. Armonk, NY). For descriptive data from the preliminary cadaveric study, mean, standard deviation (SD), and 95% confidence interval (95% CI) were calculated for ΔTU and PROM. Intra-rater reliability of VECDI and PROM measurements was assessed using data collected from three repeated measures from all eight cadaveric specimens. Investigators performing PROM and VECDI measurements were blinded to measurement values. Two separate intraclass correlation coefficients (ICC_3,1_) were used to calculate intra-rater reliability for VECDI and PROM measures. The root mean square standard deviation (RMS-SD) values were calculated for VECDI.

In order to support proof-of-concept that VECDI could detect STJ stiffness changes, the cadaveric STJ stiffness (i.e. ΔTU) and PROM values were assessed between the two mobility conditions (screw fixation versus no-screw fixation) using paired t-Tests. Two-tailed paired t-tests were performed to test for a difference between conditions of normal stiffness and screw fixation for both ΔTU and ankle complex PROM. For statistical significance, alpha was set at .05. A Pearson product moment correlation was calculated to determine the relationship between STJ VECDI values and ankle complex PROM in the same sample.

For the *in vivo* study, descriptive statistics were calculated to check for data normality. Normal distribution was considered present when at least two out of the three following criteria were met: Shapiro Wilk test p-value >.05, skewness and or kurtosis each between -2.0 and +2.0. In addition, descriptive data were calculated to summarize the sample’s demographic characteristics. Group descriptive data in terms of mean, standard deviation (SD), and 95% confidence interval (95% CI) were derived using each subject’s three-trial ΔTU average.

In order to address H1, intraclass correlation coefficients (ICC_3,1_) representing relative agreement were performed to determine intra-rater reliability of STJ stiffness (ΔTU), PROM, and MFWDiff measures and one ICC_2,3_ was performed to measure relative inter-rater reliability for stiffness measures. In accordance with other authors we considered values between .75 and .90 representing good reliability and values greater than .90 being indicative for excellent reliability [31,32]. As previously mentioned, intra-rater reliability for STJ stiffness was calculated for rater-1 using all 28 subjects and for rater-2 using the first 10 subjects. Root mean square standard deviation values (RMS-SD) were calculated to represent precision and absolute consistency of STJ stiffness (ΔTU), PROM and MFWDiff for both within-raters and between raters [33]. Intra-rater RMS-SD was calculated using each rater’s three repeated measurements. Inter-rater RMS-SD for stiffness was calculated using each raters’ mean from the three repeated measures. Pearson product-moment correlations were calculated to determine the relationship between ΔTU and PROM measured at each location.

In order to address H2, a second Pearson product-moment correlation was calculated to assess the relationship between MFWDiff and either dorsum or posterior PROM, where dorsum PROM refers to the values obtained by the ELGON that was fixed to the foot’s dorsum and posterior PROM refers to the values obtained by the ELGON that was adhered at the ankle’s posterior aspect. A third Pearson product-moment correlation was calculated to quantify the relationship between STJ stiffness (represented by ΔTU) and foot mobility (represented by MFWDiff).

In order to address a noted large ΔTU variance and further support proof-of-concept that VECDI could detect STJ stiffness changes i*n vivo*, we used a subsequent exploratory analysis to better understand data variance patterns and how those patterns affected the same relationships between ΔTU and our reference standards. To do this, we first placed each subject into one of three classification subgroups based on ΔTU dispersion from the sample mean in accordance with previous investigators [16,34]: increased stiffness (>1.0 SD below the mean), normal stiffness (from -1.0 to +1.0 SD from the mean) or decreased stiffness (>1.0 SD above the mean). Then, in order to address H3, it was necessary to establish if differences existed between those groups in order to set a precedence for further examining the relationships between ΔTU and PROM testing within the subgroups. We then used a one-way ANOVA to determine if differences existed in ΔTU among the three newly created groups. Similarly, two one-way ANOVAs (one for each PROM measure) were performed to assess for PROM differences among the three groups (α = .05). Finally, because significant differences were detected, a Pearson product-moment correlation was performed within each subgroup to assess the relationship between STJ stiffness (i.e. ΔTU) and available passive ankle-foot movement (i.e. PROM). This data dissection into three subgroups that was based on data variance from the mean allowed us to better understand how that discovered variance influenced our hypotheses testing and conclusions.

## Results

### Hypothesis 1 testing

With regards to H1, good to excellent reliability values were established for all preliminary cadaveric study measurements. Intra-rater reliability for cadaveric VECDI measurement was found to be 0.80 (95%CI = 0.60–0.92). Root mean square standard deviation was 1.34 ΔTU for both conditions together, 1.59 ΔTU for normal stiffness, and 1.03 ΔTU for simulated hypomobility. Additionally, intra-rater reliability for ankle complex PROM measurements was found to be 0.99 (95%CI = 0.98–1.0).

Paired samples t-tests comparing mean ΔTU across structural stability conditions exhibited significant differences between conditions. First, a significantly lower ΔTU at the STJ was observed during the screw fixation versus normal condition (i.e. no screw fixation; t = 4.718; p = .002). Thus, the STJs that were stabilized by screw fixation showed a greater energy transfer across the joint. Second, paired samples t-test results revealed that ankle complex PROM was significantly lower in the screw fixation versus normal condition (t = 7.683; p < .001). Results demonstrated that increases in STJ stiffness (represented by ΔTU) corresponded with decreases in overall sample ankle PROM. This was reflected by a fair correlation between ΔTU and ankle complex PROM (r = 0.660). These preliminary findings served as precedence for continuing test procedures *in vivo*, based on appreciable changes in the VECDI values between before and after STJ screw fixation, as well as respectable changes in correspondence between VECDI and PROM values.

Mean and standard deviation values for *in vivo* subject demographics, STJ stiffness (represented by ΔTU) and ankle complex PROM can be found in Table 1. All variables, with the exception of previous injuries, were considered normally distributed (Table 2). There was one subject who had 4 previous injuries, one subject that had 2 previous injuries, and 2 subjects that had 1 previous injury. The remaining 24 subjects had no previous injuries.

**Table 1:** *In vivo* subject demographics.

**Variable**	**Mean (SD)**	**Range**
**Age (y)**	25.1 (4.5)	20–36
**Height (m)**	1.7 (0.11)	1.52–1.95
**Weight (kg)**	69.1 (16.1)	478.3–113.6
**Variable**	**Frequency**	**Percentage**
**Male**	11	39.3%
**Female**	17	60.7%
**Variable**	**Maximum (Mode)**	**Range**
**Previous Injuries**	4 (0)	0–4

y = years, m = meters, kg = kilograms, SD = standard deviation

**Table 2:** Descriptive statistics for pertinent outcome data.

Variable	Mean (SD)	Range	Skewness	Kurtosis	Shapiro-Wilk
Dorsum PROM	21.63 (8.17)	8.33–38.33	0.557	-0.423	*p* = .197
Posterior PROM	22.52 (7.04)	12–41.33	0.693	0.409	*p* = .295
VECDI (ΔTU)	4.81 (1.48)	2.33–7.50	0.190	-1.150	*p* = .227
MFWDiff (mm)	7.08 (2.59)	1.39–13.98	0.306	0.985	*p* = .909

PROM = Passive Range-of-Motion; VECDI = Vibration energy with color Doppler imaging; MFWDiff = Midfoot-width-difference, ΔTU = change in color Doppler threshold units (dimension dB)

All reliability data can be found in Table 3. With regard to H1, in general, good to excellent reliability values were established for all intra-rater reliability measurements. Intra-rater reliability for VECDI measurement was found to be good for rater-1 (0.84) and rater-2 (0.76). Inter-rater reliability of VECDI was found to be poor (-3.11). Intra-rater reliability for midfoot width measurements was excellent for non-weight bearing (0.98) and for weight bearing (0.99) measurements. Additionally, intra-rater reliability was found to be excellent for dorsum (0.97) and posterior (0.97) PROM measurements.

**Table 3:** Rater reliability for all measures.

Variable	RMS-SD	ICC	ICC 95% CI	ICC Significance
VECDI Rater 1*	0.629 ΔTU	.842	.729-.917	*p* < .001
VECDI Rater 2*	0.619 ΔTU	.757	.457-.925	*p* < .001
VECDI Inter-Rater	1.737 ΔTU	-3.114	-52.4–0.158	*p* = .975
Dorsum PROM*	1.877 deg	.967	.939-.983	*p* < .001
Posterior PROM*	1.516 deg	.965	.937-.983	*p* < .001
MFW-WB*	0.604 mm	.994	.988-.997	*p* < .001
MFW-NWB*	1.076 mm	.979	.962-.990	*p* < .001

VECDI = Vibration energy with color Doppler imaging; ΔTU = change in threshold units, PROM = Passive Range-of-Motion; MFW-WB = Midfoot width–weight bearing; MFW-NWB = Midfoot width–non weight bearing; RMS-SD = root mean square of successive differences; ICC = Intraclass correlation coefficient,

*indicates intra-rater reliability

### Hypothesis 2 testing

With regard to H2, a significant positive low to moderate correlation was found between ΔTU and both dorsum PROM (*r* = .440, *p* = .019,adjusted R^2^ = 0.163) and posterior PROM (*r* = .390, *p* = .040;adjusted R^2^ = 0.12). No significant relationship was found between ΔTU and MFWDiff (*r* = .103, *p* = .603) or between MFWDiff and either dorsum PROM (*r* = .256, *p* = .188) or posterior PROM (*r* = .301, *p* = .119).

### Hypothesis 3 testing

**Fig 3:**
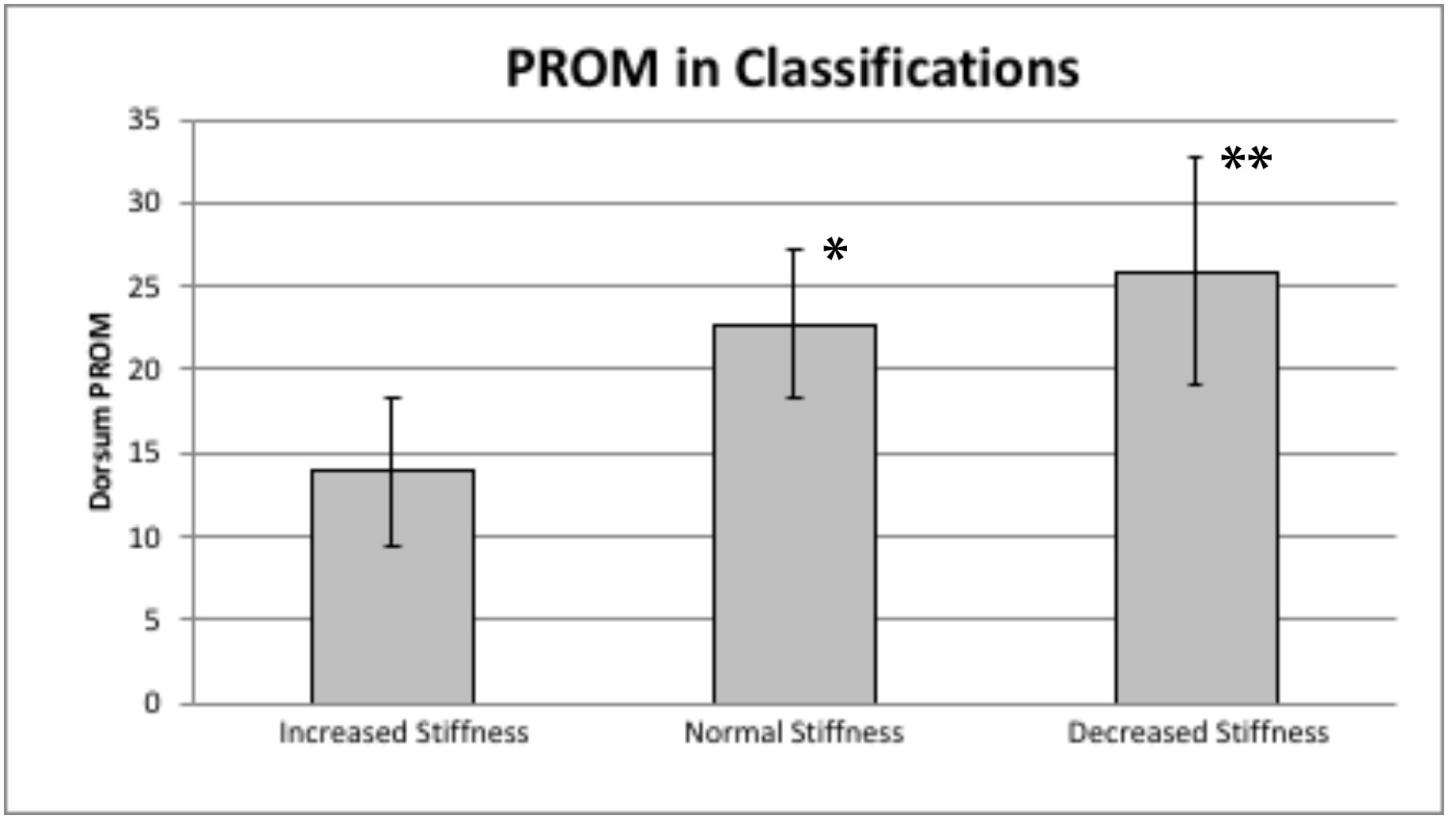
Histogram of dorsum PROM divided into classifications and measured with an electrogoniometer. Included data are mean and 95% CI (error bars). The Normal Stiffness group (*) and Decreased Stiffness group (**) mean scores each demonstrated significantly greater PROM versus the Increased Stiffness group.

The impact of subject stiffness and PROM variance on these correlations was explored to address H3 and further the proof-of-concept that VECDI can detect STJ stiffness changes. To achieve this, subjects were divided into three classifications based on ΔTU values that represented increased, normal, and decreased stiffness. As demonstrated in Table 2, the mean ΔTU value was 4.81 (SD = 1.48), resulting in a classification of increased stiffness (<3.33 ΔTU), normal stiffness (3.34–6.29 ΔTU), and decreased stiffness (>6.29 ΔTU). Six, 15 and seven subjects demonstrated increased, normal and decreased STJ stiffness, respectively. A one-way ANOVA was incorporated to objectify this classification, demonstrating a significant main effect for classification based on ΔTU [F(2,25) = 54.47,p< .001]. Post hoc tests revealed significant differences between all stiffness groups (p< .001). Additionally, a second ANOVA demonstrated a significant main effect for classification based on dorsum PROM [F(2,25) = 4.85,p = .017]. Post hoc tests revealed significant differences in dorsum PROM between increased and normal stiffness (p = .044) and between increased and decreased stiffness (p = .017), but no significant difference was found between normal and decreased stiffness (p = .620). This classification analysis can be found in Fig 3. Finally, a third ANOVA demonstrated a significant main effect for classification based on posterior PROM [F(2,25) = 3.79,p = .036]. Post hoc tests revealed a significant difference in posterior PROM between the increased and decreased stiffness groups (p = .044) but no difference was found between increased and normal stiffness (p = .061) or normal and decreased stiffness (p = .834) groups.

In response to these findings, correlations were conducted between the same variables within each group. A correlation between ΔTU and dorsum PROM revealed a significant and meaningful relationship in the increased stiffness group (*r* = .853,p = .031,adjusted R^2^ = 0.659) versus poor, nonsignificant relationships found in normal (r = -.042,p = .882,R^2^ = .002) or decreased stiffness (r = -.014,p = .977,R^2^ = .0002) groups. No significant correlations were found within the classification groups between ΔTU and MFWDiff or between ΔTU and posterior PROM (p > .05).

## Discussion

The purpose of this investigation was to establish the STJ stiffness range in asymptomatic volunteers, support the proof-of-concept that VECDI could detect differences in STJ stiffness, and examine relationships between STJ stiffness and foot mobility. The preliminary cadaveric study established our methods and provided initial support for the VECDI STJ proof-of-concept, while the *in vivo* study quantified the STJ stiffness range in asymptomatic volunteers and examined relationships between STJ stiffness and foot mobility measures, thus furthering proof-of-concept support. Finally, the *in vivo* study established ranges and limitations of VECDI intra-rater and inter-rater reliability for examining STJ stiffness changes.

Our H1 was partially supported by this study’s findings. This study demonstrated good intra-rater reliability of VECDI in this asymptomatic sample for each rater. This is consistent with our preliminary cadaveric STJ testing, as well as studies using VECDI at both the sacroiliac joint and first tarsometatarsal joint [23]. However, consistent with a previous study measuring sacroiliac joint stiffness using VECDI, inter-rater reliability was found to be poor [24]. The low inter-rater reliability may be a result of raters’ differing levels of experience using VECDI and may highlight the subjective nature of determining a cut-off point for documenting the TU values [24]. This emphasizes the need for repeated measures to be performed by one individual, as comparing values from more than one rater may not consistently reproduce measurements.

The current study’s findings reflect a novel approach to objectifying STJ stiffness, which clinicians and researchers could find useful when attempting to evaluate changes in STJ mobility. A paucity exists in the literature regarding the reliability of STJ mobility measures. Keenan and Bach [17] studied the reliability of commonly used open and closed chain clinical hindfoot mobility measures, including STJ neutral position, STJ ROM testing, neutral calcaneal stance position and resting calcaneal stance position. Similarly, they evaluated dynamic hindfoot assessment reliability using recorded barefoot treadmill walking [17,35]. They observed modest inter-tester reliability between four experienced testers for the majority of their measurements. The same investigators’ test-retest (intra-tester) results were fair-to-poor with large 95% confidence intervals during the same measurements. Moreover, intra-tester reliability for five different aspects of dynamic clinical hindfoot mobility were found poor in two studies by the same investigators [17,35]. This contrasts our good intra-rater reliability with VECDI measurements in both cadaveric and *in-vivo* samples.

Future research needs to be directed towards improving inter-rater reliability before multiple testers can be used for classification or repeated measures. Increased rigor in several factors may assist in improving inter-rater reliability. These may include increased practice together by both raters prior to initiating data collection, along with encouraging improved surface palpation skills, standardized transducer pressure, and standardized criteria for threshold points.

Our study’s results substantiate the idea that STJ stiffness and ankle complex mobility are related, as evidenced by a fair correlation between VECDI and ankle complex PROM, thus partially supporting our H2. Although intra-rater reliability for MFWDiff and both PROM measures were excellent, no correlation was found between MFWDiff and any other collected variable, including ΔTU (i.e. STJ stiffness) and ankle-foot PROM. This suggests that foot mobility as measured by MFWDiff may be unrelated to STJ stiffness and ankle-foot PROM in this asymptomatic sample. Moreover, the MFWDiff values of 7.1 (SD = 2.6) mm as measured in this sample are similar to results of a previous study demonstrating a mean MFWDiff of 9.3 (SD = 3.2) mm[16]. Contrary to our H2, the results of the current investigation do not support the presence of a relationship between STJ characteristics (including ankle-foot PROM and STJ stiffness) and foot mobility. As a result, one should not infer a single motion segment’s stiffness (e.g. at the STJ) from the clinical movement status of the entire ankle-foot system. Thus, the clinician may be inspired to further examine each joint system (such as the STJ) even when the entire ankle-foot complex does not first appear to be stiff.

**Fig 4:**
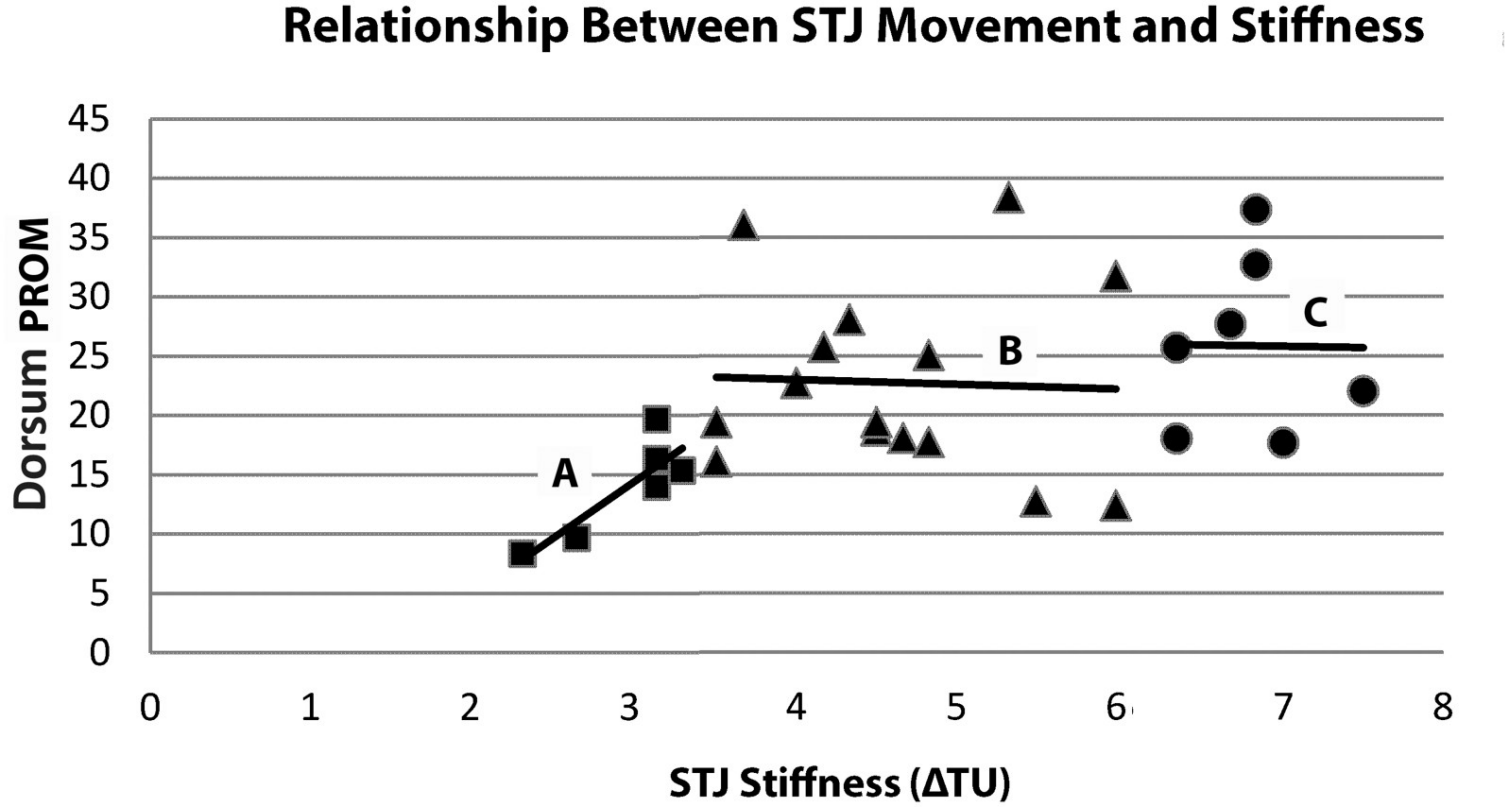
Scatter plot of STJ Stiffness (ΔTU) and dorsum PROM (degrees) values. Squares = Increased stiffness with a significant and meaningful correlation (*r* = .853,p = .031,n = 6; adjusted R^2^ = 0.659) with corresponding line-of-best-fit (A); Triangles = Normal stiffness group with a poor and non-significant relationship (r = -.042,n = 15,p = .882,R^2^ = .002) with corresponding line-of-best-fit (B); and Circles = Decreased stiffness group with a poor and non-significant relationship (r = -.014,n = 7,p = .977,R^2^ = .0002) with corresponding line-of-best-fit (C).

We were able to divide subjects into three groups based on ΔTU mean and standard deviation values. After dividing the sample, analyses results demonstrated significant differences between the three groups, thus supporting H3. Within this classification system, dorsum PROM values were significantly different between increased and normal stiffness and between decreased and increased stiffness, but no difference was found between normal and decreased stiffness. This lack of PROM difference between the normal and decreased stiffness groups can have two plausible explanations. First, given the difficult nature of clinically measuring STJ mobility, it is possible that VECDI may be a more sensitive tool for representing STJ stiffness than PROM alone. Secondly, based on a visual analysis of a simple scatter ΔTU and PROM value plot (Fig 4), it is possible that as ΔTU increases (i.e. stiffness decreases), the relationship becomes weaker. This could be due to an actual change in the relationship between stiffness and PROM as the values increase, or it may demonstrate that VECDI is a more valid measure in stiffer joints. The suggestion that PROM and stiffness may be more closely related when the joint displays increased stiffness is further supported by the significant correlation between ΔTU and PROM in the increased stiffness group but not in the normal or decreased stiffness groups.

Considering the moderate relationship between ΔTU and PROM (r = 0.440, adjusted R^2^ = 0.163), PROM may only explain approximately 16% of STJ stiffness variance. While the two measures are related to a moderate degree, using one measure to infer the other would be inappropriate since 84% of STJ stiffness variance is unexplained by PROM alone. As a result, it is important to not only assess total distance moved through PROM, but also assess the movement resistance within the available range. Since vibrations applied during VECDI assessment are very small (<0.05mm) and stay within the unconstrained region of movement (joint neutral zone), it is apparent that VECDI is not measuring PROM, but rather another non-angular characteristic influencing joint mobility, such as joint stiffness. This observation is consistent with previous investigations using VECDI at the first tarsometatarsal joint [23,29].

Multiple pathologies may present with increased STJ stiffness. Researchers have demonstrated decreased frontal plane calcaneal excursion in subjects with diabetes mellitus [11]. Rao et al [2] found a negative correlation between frontal plane calcaneal excursion and medial/lateral calcaneal plantar pressures, where increased pressure corresponded with increased susceptibility to tissue breakdown. Subtalar joint movement dysfunction may develop due to factors associated with calcaneal fractures including joint geometry changes [5] and prolonged immobilization. With these findings in mind, it is important to have a reliable measure to identify individuals who may benefit from treatment targeted at improving STJ movement status [6,11].

Future research using VECDI at the STJ should focus on assessing stiffness in subjects with suspected movement dysfunction and evaluating how these subjects fit into our reported stiffness classification. Additionally, since VECDI is not currently practical for widespread clinical use, it is necessary to use this measure to validate more frequently used manual STJ mobility assessments. Finally, VECDI could be used to experimentally measure STJ stiffness changes in response to manipulative procedures aimed at improving STJ mobility.

This investigation’s limitations should be examined. The sample used in this investigation’s *in vivo* study was asymptomatic at the time of data collection and the majority of subjects had never experienced an ankle injury. Thus, we cannot generalize our findings to individuals with present pathology. However, it was first necessary to create a classification system of ΔTU values in a normal population and establish rater reliability before attempting to measure individuals who display current lower extremity symptoms. Additionally, a convenience sample was used for our *in vivo* study, where subjects tended to be young and of healthy weight. This may limit the ability to generalize the current results to the general population, who might be of greater body mass index or elderly individuals possibly presenting with mobility restrictions due to ankle-foot joint degenerative changes. Furthermore, STJ stiffness was measured in a non-weight bearing position. While this non-weight bearing position may decrease the applicability of these findings to STJ movement characteristics in weight bearing, assessing this measure in weight bearing may create joint surface approximation, thereby increasing vibration transmission through the STJ and limiting our ability to assess joint stiffness independent of the effects of gravity. Lastly, our data collection and analyses for H2 and H3 were based only on one rater’s measurements. Furthermore, each measurement was taken multiple times by that one rater in order to use the mean value for further analyses, thus avoiding snapshot measurement values.

In conclusion, VECDI is an objectifiable instrument for measuring STJ stiffness when the same rater completes repeated measures. The VECDI data from this sample of asymptomatic subjects were normally distributed and could be separated for classification purposes into three stiffness groups based on mean and standard deviation. This classification system can be used in future research on symptomatic subjects as a basis for comparison. The modest relationship between ankle-foot PROM and STJ stiffness demonstrates that clinically PROM may not represent all aspects of joint mobility. Therefore, joint VECDI stiffness assessment should be considered as a compliment to current PROM measurement techniques.

## Supporting information

S1 Data (XLSX)
